# Explaining Obesity- and Smoking-related Healthcare Costs through Unconditional Quantile Regression

**DOI:** 10.36469/9849

**Published:** 2016-11-11

**Authors:** Bijan Borah, James Naessens, Kerry Olsen, Nilay Shah

**Affiliations:** 1 Mayo Clinic, Health Care Policy and Research Division, College of Medicine, Rochester, MN, USA; 2 Mayo Clinic, Otorhinolaryngology Dept., College of Medicine, Rochester, MN, USA

**Keywords:** unconditional quantile regression, smoking, obesity, healthcare costs, generalized linear modeling, conditional quantile regression

## Abstract

**Background:** This paper assesses obesity- and smoking-related incremental healthcare costs for the employees and dependents of a large U.S. employer.

**Objectives:** Unlike previous studies, this study evaluates the distributional effects of obesity and smoking on healthcare cost distribution using a recently developed econometric framework: the unconditional quantile regression (UQR).

**Methods:** Results were compared with the traditional conditional quantile regression (CQR), and the generalized linear modeling (GLM) framework that is commonly used for modeling healthcare cost.

**Results:** The study found strong evidence of association of healthcare costs with obesity and smoking. More importantly, the study found that these effects are substantially higher in the upper quantiles of the healthcare cost distribution than in the lower quantiles. The insights on the heterogeneity of impacts of obesity and smoking on healthcare costs would not have been captured by traditional mean-based approaches. The study also found that UQR impact estimates were substantially different from CQR impact estimates in the upper quantiles of the cost distribution.

**Conclusions:** These results suggest the potential role that smoking cessation and weight management programs can play in arresting the growth in healthcare costs. Specifically, given the finding that obesity and smoking have markedly higher impacts on high-cost patients, such programs appear to have significant cost saving potential if targeted toward high-cost patients.

## INTRODUCTION

A significant body of literature has shown that obesity and smoking, two of the most common modifiable risks for many chronic diseases, have substantial impact on overall healthcare costs.[1,2,3,4] The prevalence of obesity has been rising rapidly for a number of years and has more than doubled in the past 30 years in the United States.[5,6] This rapid rise in obesity has been associated with a substantial impact on obesity-related medical treatments and expenditures. Wolf *et al.*[7] estimated an 88% increase in the number of physician office visits resulting from obesity between 1988 and 1994. Quesenberry *et al.*[8] estimated that individuals who are moderately obese (30≤body mass index [BMI]≤34.9) and severely obese (BMI≥35) have 14% and 25% more physician visits, and 34% and 74% more inpatient days, respectively. Thompson *et al.*[9] found that obese adults (BMI≥30) have 38% more visits to primary care physicians and 48% more inpatient days per year. They also found that obese individuals had higher use of prescription drugs in general, and had much higher use of prescription drugs for diabetes and cardiovascular disease.

Using nationally representative data, two studies found the incremental annual costs of obese individuals compared to normal-weight individuals to be between 36% and 38% in 1998 dollars.[10,11] Finkelstein *et al.*[11] found that the mean incremental cost of obesity was $732, with obesity-related out-of-pocket costs estimated at $125; the corresponding obesity-related costs for Medicare and Medicaid recipients were $1,486 and $864, respectively. The economic implications of obesity, based on both healthcare and non-healthcare expenditures, are expected to worsen in the future. Wang *et al.*[12] estimated the future incremental cost of obesity in the U.S. to be between $860 and $956 billion by 2030, assuming a healthcare cost ination rate of 6%; this will be approximately 15.8% to 17.6% of the total healthcare dollars.

Smoking-related diseases constitute the leading preventable cause of death in the United States,[13] as well as a major contributor to the development and progression of many chronic diseases. The negative health effects and associated costs of smoking by employees are substantial, including both direct (healthcare costs) and indirect (including productivity loss, absenteeism, and recruitment and retraining) costs. Sturm[10] estimated that the incremental annual cost associated with ever-smokers compared to non-smokers was $230 in 1998 dollars. A systematic review by Warner *et al.*[14] found that the medical costs of smoking in the United States ranged between 6% and 8% of healthcare expenditures.

Recognizing the substantial potential for obesity and smoking to generate adverse health outcomes, and the consequent healthcare cost escalation, many U.S. employers and health plans have started offering wellness programs to reduce the prevalence of obesity and smoking.[15,16,17] A recent estimate shows that 74% of firms in the United States offering health benefits offer at least one of the following wellness programs: weight loss programs, gym membership discounts or on-site exercise facilities, smoking cessation programs, personal health coaching, classes in nutrition or healthy living, web-based resources for healthy living, or a wellness newsletter.[18] Wellness programs in the United States. have been generally shown to deliver positive return on investment.[17] However, employers often struggle to achieve a higher rate of program participation by employees in the wellness interventions; better results, both in terms of health outcomes and healthcare cost containment, can be obtained through targeted wellness programs, which specifically target employees that will benefit the most.[19] For example, subjects for whom obesity and smoking might have the largest impact on healthcare costs may benefit the most from an intervention for weight management and smoking cessation.

Previous studies assessing the impacts of obesity and smoking on healthcare costs focused primarily on the impacts on the mean cost. As a result, those studies do not shed light on the potential heterogeneity in the impacts of obesity and smoking across the healthcare cost distribution. That is, one does not know whether the extent of impact of obesity (or, of smoking) on healthcare cost for a low-cost individual (e.g., someone in the 10th percentile of the cost distribution) differs from a high-cost individual (e.g., someone in the 90th percentile). A better understanding of the distributional impacts of obesity and smoking on healthcare costs can potentially help employers develop targeted strategies for weight management and smoking cessation for their employees. This is because patients in the upper cost quantiles are expected to be different from those in the lower cost quantiles, in terms of their baseline characteristics and the severity of comorbidity profile than lower cost patients, and therefore, the former needs more aggressive intervention strategies.[20]

In order to assess the distributional impacts of obesity and smoking on overall healthcare costs, this paper implements a recently developed econometric method - unconditional quantile regression (UQR) by Firpo *et al.*[21] (henceforth FFL). The UQR approach allows one to estimate the impact of obesity and smoking at any point in the healthcare cost distribution. We also compare UQR results with the traditional conditional quantile regression (CQR) of Koenker and Bassett,[22,23] and with results from the generalized linear modeling (GLM) approach that is often used for modeling healthcare costs.[24] In addition, this paper takes advantage of two aspects of the data. The data used for this study link both healthcare payer and provider data for each subject. Provider data allow us to incorporate clinical and other measures of risk such as BMI and smoking status that are generally not available in payer (claims) data. We are able to examine longitudinal effects of obesity and smoking on healthcare costs in an insured population. Much of the previous work has focused on 1- to 2-year costs, while this paper will allow us to look at the distributional effects of costs over a 5-year period.

Note that, in order to obtain valid comparisons of the effects of obesity and smoking between UQR, CQR and GLM frameworks, obesity and smoking will be modeled as independent predictors as in Sturm.[10] Our approach might appear incongruent with prior works by Gruber and Frakes,[25] Flegal[26] and Baum.[27] However, note that there is no consensus with regard to the potential dependence between obesity and smoking. For example, Chen *et al.*[28] found that smoking does not have a long-term causal effect on body weight, and Nonnemaker *et al.*[29] could not empirically support the association between smoking cessation and weight increase. Furthermore, as reported in the Results section, we have also conducted sensitivity analyses to ensure that our modeling of obesity and smoking as independent predictors does not unduly bias the results. The plan for the rest of the paper is as follows. Section 2 introduces the UQR method. Since UQR is discussed in detail by FFL,[21,30] we provide only a brief summary of the UQR methodology. Section 3 discusses the data for this study. Section 4 provides model results, and compares them with the results obtained from standard CQR and GLM framework. We discuss the implications of the study results in Section 5, and provide the conclusions of the study in Section 6.

### UNCONDITIONAL QUANTILE REGRESSION

The UQR method as introduced recently by FFL[21] provides a novel framework for assessing the impact of the distributional changes of dependent variables on marginal (unconditional) quantiles of the outcome of interest. One appealing feature of the commonly used ordinary least square (OLS) regression framework is that it helps quantify the impact of an independent variable on the conditional mean of the outcome variable, which in turn, through the law of iterated expectations, provides consistent estimate of such impact on the unconditional population mean outcome. However, there arise situations where it is critical to understand the impact of the explanatory variables across different parts of the outcome variable. For example, it is not only important to understand the impact of obesity and smoking on average healthcare costs, but in order for the policy maker to devise effective healthcare cost-reduction strategies through prevention or other incentive programs, it is important to assess the impacts of obesity and smoking beyond mean healthcare costs. Specically, it will be insightful to evaluate the impact of obesity or smoking in the upper tail of the cost distribution, as the latter is known to be skewed toward the right. Traditionally, this issue has been addressed in the literature through CQR.[22,23] CQR does quantify the impact of an explanatory variable on the outcome variable across its entire distribution. However, CQR does not provide a consistent estimate of the population quantile.[21] This makes it difficult to answer questions such as 'what is the impact on median healthcare costs when the proportion of smokers is reduced by a particular amount?'. As highlighted in FFL,[21] the UQR methodology helps unravel the distributional impact of an explanatory variable on the outcome of interest at the population level.

FFL's UQR method makes use of inuence function (IF), a widely used concept in robust estimation literature.[31] The IF of a statistic refers to the inuence of an individual observation on that statistic. Recentered influence function, or RIF, is obtained by adding the statistic to its influence function. For the $T^{th}$ conditional quantile, $q_T$, of the distribution of the outcome variable $Y$, the influence function $IF(Y, q_T)$ is given by $(\tau - I\{Y \leq q_\tau\})/f_Y(q_\tau)$, where $I\{.\}$ is an indicator function and $f_Y(.)$ is the probability density function (pdf) of $Y$. Thus, by denition, the RIF of $q_T$ is $RIF(Y: q_T) = q_T + IF(Y, q_T)$.

<u>RIF-Regression Model</u>: FFL defined RIF-regression model for the statistic $v$ as the conditional expectation of the RIF of $v$, expressed in terms of the explanatory variables $X$:

$$E[RIF(Y;v)|X] = m_v(X). \tag{1}$$

When the statistics of interest are quantiles of the outcome distribution, FFL have shown that $E[RIF(Y; q_T)|X] = m_T(X)$ can be interpreted as the UQR because its average derivative $E[m'_T(X)]$ can be shown to be equal to the marginal effect on the $T^{th}$ unconditional quantile of $Y$. In terms of implementation of the UQR method, the steps are literally similar to the OLS implementation. For the $T^{th}$ quantile, $q_T$, the dependent variable is its RIF given by:

$$RIF(Y; q_\tau, F_Y) = q_\tau + (\tau - I\{Y \leq q_\tau\})/f_Y(q_\tau). \tag{2}$$

The components of the dependent variable in equation (2) can be easily computed. The first term on the right-hand side of equation (2) is the $T^{th}$ sample quantile of $Y$; $f_Y(q_T)$ is the pdf of $Y$ estimated at $q_T$ through kernel or other methods; and $I\{Y \leq q_\tau\}$ is a dummy variable, indicating whether the outcome variable is less than $q_T$. Once this dependent variable is constructed, the next step is to run an OLS regression of this dependent variable on the explanatory variables.

FFL's[21] UQR framework complements the existing literature on quantile function estimation.[32,33,34] However, in contrast to these authors who estimated quantile functions in presence of endogenous regressors, the UQR framework considers only exogenous regressors.

#### Unconditional Quantile Partial Eects

Unconditional quantile partial effect (UQPE) measures the effect of an explanatory variable on the unconditional quantile of the outcome variable in a sense similar to Wooldrige's unconditional average partial effect defined as $E[dE[Y|X]/dx]$.[35] FFL[21] defined U QP E(τ) of an explanatory variable $X$ on the $T^{th}$ quantile of the outcome variable $Y$ as follows:

$$\alpha(\tau) = c_{1\tau} \int \frac{dPr[Y > q_\tau | X = x]}{dx} dF_X(x), \tag{3}$$

where the term under the integral sign is the marginal effect from the probability response model:

$$Pr[Y > q_\tau | X = x], \text{ and } c_{1\tau} = 1/f_Y(q_\tau).$$

As elaborated in FFL,[21] in order to implement the estimation of the UQPE(τ) in equation (3), the following three components need to be estimated:

1. The quantile $q_\tau$, which can be estimated by the $\tau^{th}$ sample quantile, as defined in Koenker and Bassett;[22]
2. The pdf of the unconditional distribution of $Y$ at $q_\tau$, which can be estimated by kernel density estimation method; and
3. The average marginal effect $E(dPr[Y > q_\tau | X])/dX$, which can be estimated from the RIF-regression (also known as RIF-OLS Regression) described in equation (2). The assumption required for consistency of RIF-OLS estimates is that the $Pr[Y > q_\tau | X]$ is linear in $x$. Note that FLL[21] suggest two more methods; however, since those methods were shown to be complementary, we focus only on RIF-OLS regression.

The estimate of UQPE for a binary explanatory variable can be obtained by estimating $E(Pr[Y > q_\tau | X = 1]) - E(Pr[Y > q_\tau | X = 0])$. In this case, the UQPE may be interpreted as the impact of a small change in the probability $p = Pr[X = 1]$, instead of the effect of a small locational shift for a continuous variable.[21] FFL established that UQPE can be expressed as the weighted average of conditional quantile partial effects (CQPEs) estimated from CQR. They also discuss asymptotic properties of UQPE.

### DATA

The data for this study came from multiple sources. The study sample was selected using health insurance enrollment files for Mayo Clinic employees and dependents. Only the employees and dependents fully covered by the health plan were included. Healthcare costs were calculated using the medical and pharmacy claims files. Costs of all health care services included in the health plan claims for the study period were included in our analyses. Data were extracted from medical and pharmacy claims that capture each unit of service paid (i.e., an outpatient visit, prescription, inpatient day). For each service, the paid amount (including plan and enrollee components) was used to capture total costs for each set of services.

This initial dataset was then supplemented by Mayo Clinic electronic health records (EHR) for some key variables: BMI, smoking status, race, marital status and education level. BMI was calculated based on the height and weight measured at the office visit during the baseline timeframe. Smoking status, marital status, race and education level were reported annually in the current visit information that each patient was required to complete. We then combined data from both healthcare provider (Mayo Clinic) and the payer (health insurance plan providor for employees and dependents). This significantly enriched the data quality since health plan claims data provide accurate information on healthcare utilization in an insured population, but they may not capture information on smoking status, BMI, race or marital status. The healthcare provider, conversely, may capture the information on the above variables quite accurately; however, the provider data may not capture healthcare resource utilization data accurately, particularly when subjects receive care from multiple providers. The ability to combine provider and payer data in this study is unique in the sense that it is known to be very difficult to obtain data for the same subjects from these two sources. The protocol for this study was approved by Mayo Clinic Institutional Review Board for human subject research.

Study subjects were 18 years or older as of the year 2001 and continuously enrolled in the health plan from 2001 through 2007 (study period). The period from 1999 to 2002 was considered the baseline period, during which baseline characteristics for the subjects were captured. The outcome variable, all-cause healthcare cost, was assessed for the 5-year follow-up period: 2003 through 2007. Some key independent variables were constructed as follows:

- BMI: The World Health Organization (WHO) definition of BMI was adopted, defined as weight in kilograms (kg) divided by height in meters (m) squared or kg/m2.[36] In order to construct the BMI measure, weight for each subject was captured from EHR during the baseline period; the weight closest to 01/01/2001 was finally used. Information on height was also captured from EHR during the entire study period, as it is reasonable to expect only negligible changes in height for the adult subjects included in the study. However, the height measurement closest to 1/1/2001 was taken for BMI measurement. For pregnant women during the study period, weight measurements collected 6 months prior to and 6 months following delivery date were discarded. For our analysis, BMI was divided into five categories, with the obese category divided into two subcategories as follows: underweight (BMI<18.50), normal weight (18.5≤BMI<24.99), overweight (25.00≤BMI<29.99), obese (30.00≤BMI<39.99), and morbidly obese (BMI≥40).
- Smoking: Whether a study subject was a current smoker or not was extracted from self-reported measure in the patient-provided information sheet of the EHR during the baseline period. The "smoking status" closest to 01/01/2001 was included in the analysis.

- Comorbidities: A classification of comorbidities developed within Mayo Clinic was used to define baseline comorbidities.[37] This classification takes the chronic comorbidity codes from Hwang *et al.*[38] as a starting point, and for codes not classified by this method, the Agency for Healthcare Research and Quality (AHRQ) Clinical Classification Software (CCS) for International Classification of Disease 9th Revision Clinical Modification (ICD-9-CM) diagnosis codes were used.[39] All comorbidities that apply to the classification method reported between 1999 and 2002 (baseline period) were utilized.

Other variables used in the study are described in Table 1. The analytic dataset for this study was constructed using SAS version 9.1,[40] while the actual analyses were carried out in Stata version 11.0.[41] Besides modeling obesity- and smoking-related costs using the UQR method, we also modeled the same using two traditional methods: CQR[22,23] and GLM.[42] Assuming a linear specification in explanatory variables for conditional quantiles, the $\beta$'s from CQR refer to conditional quantile partial effects (CQPEs).

Average partial effects from the GLM regression (GLM-APE) were also estimated as GLM, a commonly used framework for modeling healthcare costs.[24] The juxtaposition of UQR results with those of CQR and GLM is expected to provide additional insights on the UQR model *vis-a`-vis* traditional CQR and GLM models. Standard errors for the estimated UQPEs, CQPEs, and GLM-APEs were all based on 500 bootstrap replications.

CQR and GLM models were estimated by using Stata's in-built commands sqreg and glm, respectively, while UQR model was estimated by Stata macro rifreg made available in the following website: http://faculty.arts.ubc.ca/nfortin/datahead.html.

### RESULTS

#### Baseline Characteristics

The final study sample included 19,492 subjects. Table 1 provides summary statistics for the baseline characteristics (mean and SD for continuous variables, and number [n] and percent [%] for categorical variables). The average age of subjects included in the sample was 40 years (SD=9.47 years). The average (SD) baseline healthcare cost was $15,591 ($33,211). The proportion of female subjects in the sample was 63%. Smokers comprised 32% of the sample. There was a substantially higher proportion of White subjects (97%) in the sample.

Approximately 79% of the sample reported being married while 16% reported being single and 5% were divorced. Distribution of the sample across the weight categories was as follows: normal weight (30%), underweight (4%), overweight (34%), obese (19%) and morbidly obese (13%). Approximately 2% of the sampled subjects had some high school education, 19% were high school graduates, 39% had some college education, 19% had a college degree and 21% had a post-graduate degree.

**Table 1.** Baseline Characteristics (N=19 492)

| Variable | Mean | SD |
|---|---|---|
| Age | 40.35 | 9.47 |
| Baseline Cost ($) | 15592 | 33211 |
| Logarithm of Cost | 8.85 | 1.51 |
| | **n** | **%** |
| Female Gender | 12185 | 62.51 |
| Whether Smoker | 6152 | 31.56 |
| **Race Category** | | |
| White | 18838 | 96.64 |
| Black | 104 | 0.53 |
| Asian/Native Hawaiian | 321 | 1.65 |
| Other | 229 | 1.17 |
| **Marital Status** | | |
| Married | 15308 | 78.53 |
| Single | 3109 | 15.95 |
| Divorced | 995 | 5.1 |
| Widowed | 80 | 0.41 |
| **BMI Category** | | |
| Normal | 5898 | 30.26 |
| Underweight | 812 | 4.17 |
| Overweight | 6540 | 33.55 |
| Obese | 3672 | 18.84 |
| Morbidly Obese | 2570 | 13.18 |
| **Education Level** | | |
| Some High School | 353 | 1.81 |
| High School Graduate | 3701 | 18.99 |
| Some College | 7642 | 39.21 |
| College Graduate | 3701 | 18.99 |
| Postgraduate Studies | 4095 | 21.01 |
| **Baseline Comorbid Conditions** | | |
| Malignancy | 760 | 3,9 |
| Thyroid Problem | 1598 | 8.2 |
| Diabetes | 783 | 4.02 |
| Hyperlipidemia | 4936 | 25.32 |
| Substance Abuse | 351 | 1.8 |
| Valvular Heart Disease | 553 | 2.84 |
| Hypertension | 3384 | 17.36 |
| Artherosclerosis | 733 | 3.76 |

**Table 1.** Baseline Characteristics (N=19 492) - Continued

| Variable | n | % |
|---|---|---|
| **Baseline Comorbid Conditions—Continued** | | |
| Dysrhythmia | 1215 | 6.23 |
| Cerebrovascular Disease | 211 | 1.08 |
| Asthma | 1888 | 9.69 |
| Esophageal Disorders | 427 | 2.19 |
| Diverticulitis | 833 | 4.27 |
| Ovarian/Uterine/Reproductive Problems | 3258 | 16.71 |
| Ulverative Colitis | 485 | 2.49 |
| Rheumatoid Arthritis | 241 | 1.42 |
| Osteoarthritis | 1984 | 10.18 |
| Back Pain | 1211 | 6.21 |
| Congenital Disorder | 958 | 4.91 |
| Glaucoma | 765 | 3.92 |
| Central Nervous System Disease | 795 | 4.08 |
| Other Heart Disease | 266 | 1.36 |
| Migraines | 1749 | 8.97 |
| Allegry | 4794 | 24.59 |
| Liver Disease | 310 | 1.59 |
| Behavior Problem | 995 | 5.1 |
| Anxiety | 1515 | 7.77 |
| Personality Disorder | 606 | 3.11 |
| Depression | 3405 | 17.47 |
| Other Mental Disease | 277 | 1.42 |

Table 1 also includes the summary statistics for the baseline comorbid conditions. We considered only those comorbid conditions with prevalence rates of 1% or higher. Hyperlipidemia, hypertension, allergy, depression and ovarian/uterine/reproductive problems had the highest prevalence rates (10% or higher).

The baseline characteristics for each of the four cost quartiles are also provided in the Appendix (see Table A.1). Note high SDs for the baseline overall mean cost in Table 1, and the baseline mean costs for each of the quartiles in Table A.1. They reflect high dispersion and extreme right-skewness in the cost variable that is typical of healthcare cost data. Although descriptive, important trends with regard to the distribution of weight categories and smoking status by the cost quartiles emerge. The proportions of obese and morbidly obese subjects increase from 17% and 8% in the lowest cost quartile to 22% in the highest cost quartile in both weight categories. Similarly, the proportion of smokers increases from 26% in the lowest cost quartile to 39% in the upper-most cost quartile. The prevalence of baseline comorbid conditions increases in a monotonic fashion as one moves from the first to the fourth cost quartile.

#### Descriptive Statistics for Overall Healthcare Costs in the Follow-up Period

The 5-year mean overall healthcare cost, the primary outcome variable, was $40,812 (SD: $63,927), while the median cost was $24,199. As expected, the cost variable is highly skewed towards the right tail, indicating presence of some very high-cost subjects. The latter is typical of healthcare cost data reflecting the fact that a small proportion of patients may have disproportionately high utilizations, and therefore they need to be accounted for in any effort to model health care cost.[43] Traditional mean-based approaches are overly influenced by the presence of such extreme observations. Both UQR and CQR frameworks, conversely, are much less amenable to outliers, which justify the use of these methods over mean-based methods.

The average cost of a smoker was $49,222 (SD: $75,918) compared to $36,934 (SD: $57,150) for a non-smoker. The unadjusted difference of $12,288 in overall healthcare costs between smokers and non-smokers was statistically significant ($p<0.001$). The unadjusted 5-year overall healthcare cost exhibited a monotonic trend as one moves from normal weight category to morbidly obese category. For example, the average 5-year overall healthcare cost for normal weight subjects was $35,076 (SD: $62,880), while for obese and morbidly obese subjects, the corresponding figures were $45,300 (SD: $65,477) and $59,308 (SD: $85,309), respectively.

**UQR Results**

Note that we modeled obesity categories and smoking as independent predictors in the UQR, CQR, and GLM frameworks. In order to ensure that our approach does not bias the results, we first assessed the correlation coefficients between obesity categories and smoking. The maximum correlation found was 0.03, implying that there was a only negligible linear relationship between obesity and smoking. We further conducted sensitivity analyses, including interaction terms between smoking and obesity categories. However, we did not find that those interaction terms were predictive of the outcome. Due to space constraints these sensitivity analyses are not provided in the paper, but they are available on request.

Table 2 presents UQPE estimates or the incremental effects from the UQR, which were estimated at 10th, 20th, ..., 90th percentiles of the healthcare cost distribution. The final UQR models for those quantiles included variables described in Table 1, square of the age variable, and the interaction between age and gender. Separate binary indicator variables for baseline comorbid conditions were used in the model. While UQPEs for other dependent variables are also presented, we focus our discussion only on the UQPEs of obesity and morbid obesity compared to normal weight category, and UQPEs of smokers vs. non-smokers.

As seen in Table 2, the impact of obesity and morbid obesity increases significantly as one moves from the left tail (lower cost quantiles) to the right tail (higher cost quantiles) of the cost distribution. Consider first the obesity category compared to the normal weight category.

The UQPEs at 20th, 50th (the median) and 90th percentiles were $905 ($p<0.05$), $2,954 ($p<0.01$) and $8,016 ($p<0.05$), respectively. A similar trend was observed for the effects of morbid obesity across different quantiles of the cost distribution; more specifically, the impact of morbid obesity appeared to be a monotonic function of the $\tau^{th}$ quantile of the cost distribution, where $\tau \in \{10, 20, ..., 90\}$. Note, however, that the impacts of morbid obesity were considerably higher than those of obesity at the corresponding quantiles.

At the 50th percentile (median) of the cost distribution, the impact of morbid obesity was $4,559 ($p<0.01$), while the impact at the 90th percentile was $33,134 ($p<0.01$). These results underscore that obesity is significantly associated with healthcare costs, and that the impact of obesity is substantially higher in the upper quantiles of the cost distribution than in the lower quantiles. This heterogeneity of impact of obesity on healthcare costs would not have been revealed from traditional (conditional) mean-based approaches such as the GLM framework. This issue will be explored further in the next sub-section.

The UQPEs of smoking at different quantiles of the cost distribution exhibited a similar pattern - the impact of smoking on overall healthcare costs increased monotonically from the lower to the upper tail of the cost distribution. At the 10th percentile, the impact was $1,172 (p<0.01), which increased to $2,846 (p<0.01) at the 50th percentile, and finally at the 90th percentile, the impact was as high as $20,011 (p<0.01).

**Table 2.** Unconditional Quantile Partial Effects (UQPEs)

| | Q10 | Q20 | Q30 | Q40 | Q50 | Q60 | Q70 | Q80 | Q90 |
|---|---|---|---|---|---|---|---|---|---|
| Age | 234† | -60 | -402* | -481* | -781* | -722* | -529‡ | -95 | 131 |
| Female | 16666* | 18048* | 21259* | 23501* | 23846* | 24761* | 24136* | 18539* | -3986 |
| Age Squared | 2‡ | 6* | 11* | 12* | 15* | 15* | 14* | 10 | 6 |
| Age*Female | -333* | -353* | -424* | -467* | -470* | -503* | -522* | -417* | 96 |
| Log Cost | 1903 | 2552* | 2931* | 3537* | 3981* | 4870* | 5950* | 7923* | 10482* |
| **Marital Status (Reference: Married)** | | | | | | | | | |
| Single | -171 | -67 | -334 | 328 | 941 | 2159† | 1508 | 5280* | 8758* |
| Divorce | 571† | 198 | -25 | -224 | 539 | 1049 | 2854‡ | 4310 | 753 |
| Widowed | -100 | 998 | -177 | 1206 | 2541 | 4824 | 7082 | 5591 | 15976 |
| **Race Category (Reference: White)** | | | | | | | | | |
| Black | 853 | -756 | -1258 | -2207 | -1259 | -954 | -2382 | 2052 | 587 |
| Asian | -562 | -795 | -513 | -1501 | -1860 | 536 | -1180 | -3001 | -6000 |
| Others | 2195* | 1381 | 2455‡ | 2882‡ | 2030 | 2492 | 2432 | 6036 | 3808 |
| **Education Level (Reference: Post-Graduate Studies)** | | | | | | | | | |
| Some High School | -2191† | -2793* | -2867* | -2139‡ | -1989 | -1644 | -1072 | -1441 | -5261 |
| High School Graduate | -1121 | -1216 | -1255 | -1432* | -1803 | -1621† | -1593 | -2627 | -7248† |
| Some College | -850* | -944* | -981* | -1112† | -1613* | -956 | -166 | -104 | -2478 |
| College Graduate | -716† | -727† | -850‡ | -908‡ | -1129‡ | -831 | -1227 | -3725† | -5870† |
| **Weight Category (Reference: Normal Weight)** | | | | | | | | | |
| Underweight | -992‡ | -884 | 759 | 345 | -406 | 293 | 2340 | 991 | -611 |
| Overweight | -120 | 198 | 757† | 538 | 1191† | 1949* | 2752* | 3310† | -1088 |
| Obese | 234 | 905† | 1452* | 1999* | 2954* | 4282* | 6302* | 9899* | 8016* |
| Morbidly Obese | 248 | 1005* | 2189* | 3231* | 4559* | 7897* | 14057* | 20292* | 33134* |
| Smoker | 1172* | 1690* | 1984* | 2533* | 2846* | 4200* | 6195* | 9133* | 20011* |

*p≤ 0.01; †p ≤ 0.05; ‡p ≤ 0.10; Baseline comorbid conditions enlisted in Table 1 were also adjusted; UQPEs were based on 500 bootstrap replications.

### UQPEs Compared to CQPEs and GLM-APEs

The comparison between UQPE, CQPE and the GLM-APE for obesity, morbid obesity, and smoking on healthcare cost are provided in Table 3. The GLM model assumed a gamma distribution and logarithmic link function.[24] While Table 3 provides the comparison for only 10th, 50th, and 90th quantiles of the cost distribution, Figure 1 captures the impacts of obesity, morbid obesity, and smoking on cost from the three frameworks, respectively, with UQPEs and CQPEs estimated at 10th, 20th, ..., 90th quantiles.

**Table 3.** Comparison of UQPE, CQPE and GLM-APE of Obesity and Smoking on Costs

| | Q10 | | Q50 | | Q90 | | |
|---|---|---|---|---|---|---|---|
| | **UQR** | **CQR** | **UQR** | **CQR** | **UQR** | **CQR** | **GLM** |
| Weight Categories (Reference: Normal Weight) | | | | | | | |
| Underweight | -992 ‡ | -153 | -406 | 449 | -611 | -723 | 633 |
| Overweight | -120 | -82 | 1191† | 929† | -1088 | 3159 ‡ | 715 |
| Obese | 234 | 155 | 2954* | 2665* | 8016* | 9602 | 5542* |
| Morbidly Obese | 248 | 565 | 4559* | 6795* | 33134* | 22061* | 11121* |
| Smoker | 1172* | 897* | 2846* | 3206* | 20011* | 14901* | 8501* |

*p≤ 0.01; †p ≤ 0.05; ‡ p ≤ 0.10; Baseline covariates in Table 1, age squared and age-gender interaction were also adjusted; UQPE - Unconditional Quantile Partial Effect; CQPE - Conditional Quantile Partial Effect; GLM-APE - Generalized Linear Model Average Partial Effect; Partial effects were based on 500 bootstrap replications.

**Figure 1. Effects of Obesity and Smoking on Costs - Comparison Between UQR, CQR and GLM**

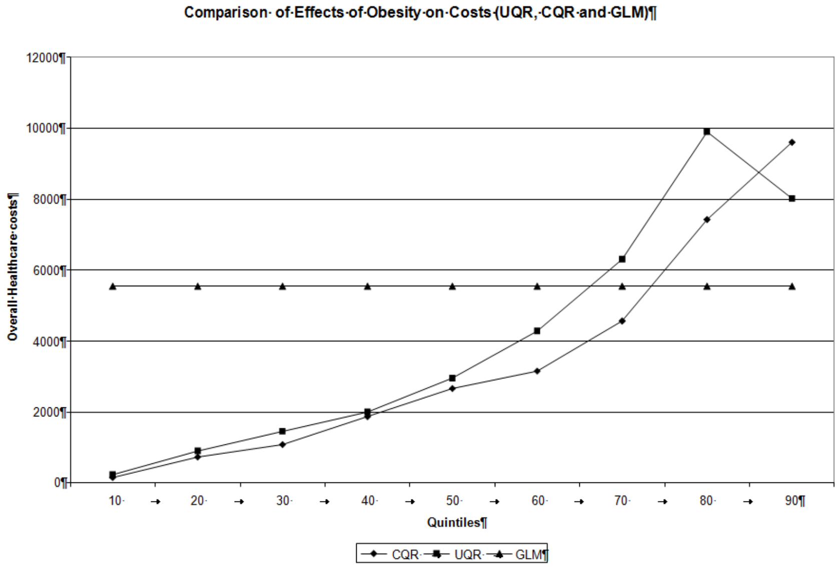

**Panel I**

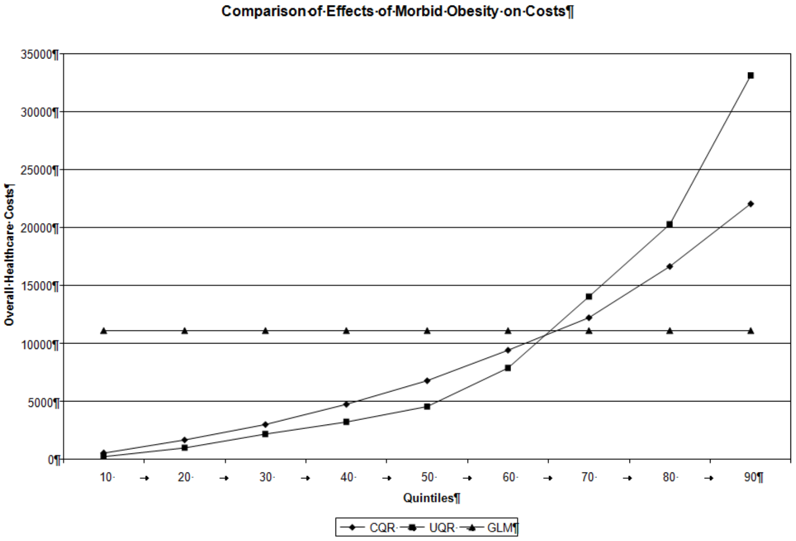

**Panel II**

**Figure 1.** Effects of Obesity and Smoking on Costs - Comparison Between UQR, CQR and GLM - Cont.

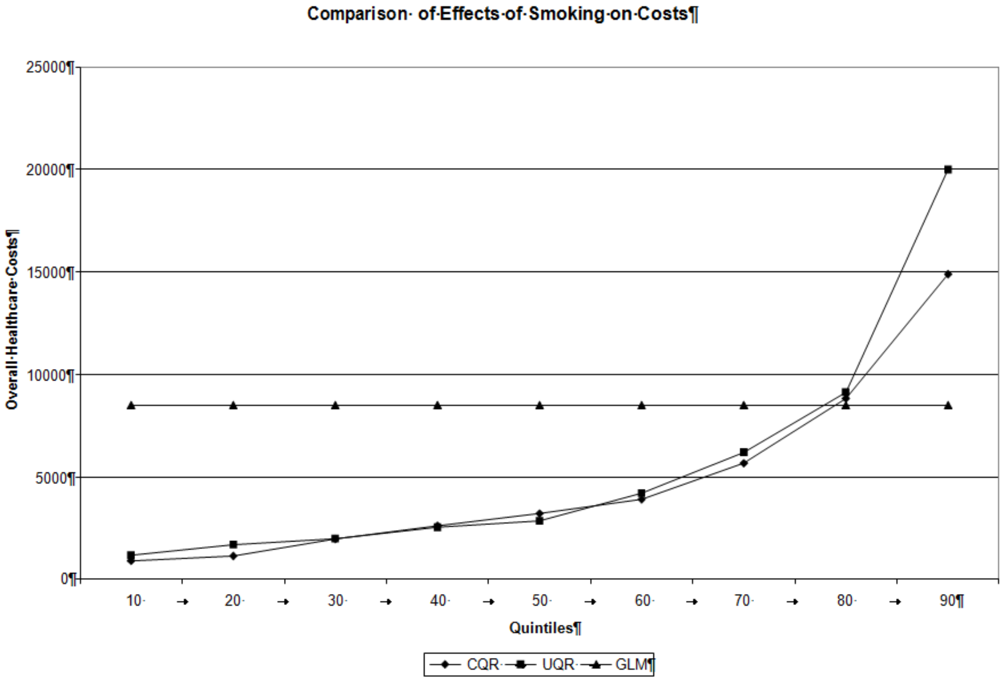

**Panel III**

The average partial effect of obesity on healthcare cost using the GLM framework was \$5,542 ($p<0.01$), which can be interpreted as the impact of obesity on the conditional mean healthcare cost. As seen from Table 3 and Figure 1, UQPEs and CQPEs unravel the intensity of the impact across different parts of the cost distribution. This complements the information gained from GLM.

Figure 1 also demonstrates the difference in the estimated effects of obesity and smoking from CQR and UQR frameworks. As seen from Panel I of Figure 1, which provides the effects of obesity on overall healthcare cost distribution, unconditional effects or UQPEs were monotonically increasing in all but the 80th cost quantile onwards. The conditional effects or CQPEs remained monotonically increasing throughout the entire cost distribution. Moreover, the two sets of effects were very similar in absolute value in the lower part of the cost distribution (below the median cost), while the difference in the estimated effects from CQR and UQR increased substantially in the upper tail of the distribution (above the median cost). The decline of UQPE at the 80th quantile, which eventually became significantly lower than CQPE (\$8,016 vs. \$9,602) at the 90th quantile, potentially reflects that UQPE is a weighted sum of the CQPEs.[21]

It is also important to emphasize the distinct advantages that the UQR estimates provide over the standard CQR framework. Under CQR, the fact that CQPE at the 90th percentile is higher than that at the 50th percentile is simply a reflection of the fact that obesity reduces within-group dispersion in costs, where "group" comprises subjects with the same values of the covariates $X$ other than the covariate indicating obese category.[21,44] This, however, does not allow us to answer important policy questions such as the impact of obesity on the overall healthcare cost dispersion measured by the difference between the 10th and 90th quantiles of the unconditional cost distribution. The CQPEs that one estimates from the standard CQR framework has an important limitation in that they do not average up to the unconditional population counterparts. This is why one has to rely on the UQPEs that estimate the effects of covariates of interest (e.g., effect of obesity) on the overall cost distribution.

Note also that besides capturing "within-group" effects of CQR described above, UQR also captures

"between-group" effects, which is driven by the fact that obesity increases conditional mean healthcare costs for obese subjects compared to normal weight subjects.[21] The extent of these two within- and between-group effects and the resulting net effect is largely an empirical question. In our specific application, it is obvious that these two effects go in tandem in the upper quantiles, resulting in larger impacts in higher cost quantiles than in lower cost quantiles.

The results for the morbidly obese category may be interpreted similarly. The effects of morbid obesity on overall healthcare costs from both CQR and UQR monotonically increased from lower to upper quantiles of the cost distribution (Table 3 and Panel II of Figure 1). However, note that UQPEs started out smaller than CQPEs in the lower cost quantiles, but around the 65th quantile UQPE became higher than CQPE and continued this trend for the rest of the upper cost quantiles. At the 90th quantile, the estimated UQPE was substantially higher than CQPE ($33,134 vs. $22,061).

As seen in Panel III of Figure 1, the effects of smoking, as measured in the CQR and UQR frameworks are very similar for most of the cost quantiles, except at the 80th quantile, where UQPE started increasing at a much higher rate than CQPE. At the 90th quantile, as shown in Table 3, UQPE of smoking was substantially higher than CQPE ($20,011 vs. $14,901).

The GLM-APEs have been overlaid in each of the three panels of Figure 1, showing the effects of obesity, morbid obesity and smoking on overall healthcare costs and illustrates the incremental benefit of UQR and CQR over the traditional GLM framework. Besides showing the heterogeneity of impacts, the juxtaposition of GLM-APEs with UQPEs also demonstrates the extent of overestimation of the effects of obesity in the lower quantiles (below the 65th quantile) and the extent of underestimation in the upper quantiles (above 65th quantile).

**Sensitivity Analyses**

We conducted several sensitivity analyses to assess the robustness of our findings on several dimensions, as described in the following sub-sections.

*Follow-up Period Duration*

Our study considered a 5-year follow-up period to assess the effects of obesity and smoking on long-term (5-year) healthcare costs. However, the 5-year period might be considered long, as some of the underlying comorbid conditions might change and/or the subject's obesity category or smoking status may change as well. Therefore, we estimated the impacts of obesity and smoking on healthcare costs just for the first year of follow-up and assessed whether the results exhibited a pattern similar to our main results (Table 2).

The results for the first year of follow-up are provided in Panel I of the Appendix Table A.2. The overall direction of the effects of obesity and smoking categories are similar to the main results in Table 2. In terms of the absolute size of the effects, the effects of obesity and smoking for the 1-year follow-up period (Table A.2) appear to be the scaled-down versions of what were observed for the 5-year follow-up period (Table 2). These results provide the sense of assurance that our main results in Table 2 are not overly impacted by the longer follow-up period.

*Impact of Gender*

Since healthcare utilization patterns (and consequently the associated costs) have been shown to be different between males and females,[45] we considered UQR analyses separately for males and females. The results are documented in Panels II and III of Appendix Table A.2, respectively. As evident from the results, the impacts of obesity and morbid obesity on overall healthcare costs were higher for female than male subjects from the 30th percentile onwards. This is in line with Finkelstein *et al.*,[46] who also found that obese women had higher impact on healthcare costs than obese men. Smoking, on the other hand, appeared to have impact on healthcare cost in the opposite direction; as one moves from the lower to the upper tail of the cost distribution, the impact is higher for male subjects than female subjects.

### DISCUSSION

This paper examined the distributional effect of obesity and smoking on healthcare spending over a 5-year period. While there has been much work on incremental healthcare costs associated with obesity and smoking, the extant literature primarily concentrates on the effects of obesity and smoking on mean costs. The standard analytic framework of mean-based analysis does not reveal the potential heterogeneous impacts of obesity and smoking across different parts of the cost distribution. The standard CQR may shed light on heterogeneity of impacts; however, unlike the conditional mean that averages to the population mean, conditional quantiles estimated through CQR do not average to their population counterparts, and therefore important policy questions such as assessing the impacts of obesity and smoking on the overall healthcare costs cannot be addressed using the standard CQR. In order to get a better and more policy-relevant understanding of the distributional effects, we used the recently developed methodology of UQR to assess the impacts of smoking and obesity on overall healthcare costs.

The results show that obesity and smoking have small impacts on healthcare costs at the 10th and 20th percentile; however, the effects on healthcare spending are substantially larger at the 80th and 90th percentiles. The effects of morbid obesity in the upper tails of the distribution are very large compared to those in the lower tail of the cost distribution. In addition, estimated impacts were generally higher for the CQR compared to the UQR until the 60th percentile and then stayed lower than the UQR for the rest of the distribution in the upper tail. The GLM results highlight that the effect of obesity, morbid obesity, and smoking on mean costs would be substantially over- and under-estimated in the lower and upper quantiles, respectively, compared to UQR.

#### Advantages of UQR Over CQR and GLM

The study demonstrates the advantages of assessing the effects of obesity and smoking through UQR compared to CQR, and the more traditional GLM approach for modeling healthcare costs. While the estimated conditional and unconditional effects of obesity and smoking on costs were generally similar in the lower quantiles of the cost distribution for our specific application, they differ substantially in the upper cost quantiles. Note that since conditional quantiles do not average to their population counterparts,[21] the estimates of CQPEs obtained from CQR may not be interpreted as the marginal effects of obesity or smoking on the corresponding unconditional quantiles (e.g., median, 90th percentile) of healthcare cost distribution, holding everything constant. The differences in the estimated conditional and unconditional effects, particularly in the upper quantiles, are testimonies to the fact that UQPEs and CQPEs may differ substantially.

A practical advantage of UQR over CQR arises when an intervention is planned for very high-cost patients, which will coordinate care between different providers so that patients' health outcomes are improved and healthcare costs are reduced through efficient delivery of care. Our hypothetical example resembles the "medical home" model in the U.S. that delivers well-coordinated care for specific patient populations including chronic disease patients.[47] Now, consider that the primary qualifying criterion for a patient to be included in the intervention is that his 5-year healthcare costs falls above the 80th percentile of the cost distribution in the target population or above a fixed high-cost threshold, say $X. The potential limitation of CQR as a distributional tool is that the high-cost threshold of $X may fall in different quantiles of the cost distribution, depending on the characteristics of the patient. The 80th quantile for some patients with specific characteristics may fall well below the high-cost threshold of $X. UQR, however, does not have this limitation, as UQPEs are marginal effects on the unconditional cost distribution and, thus, the influence of individual covariates are integrated out before arriving at these effect estimates.

Our study also underscores the heterogeneous effects of obesity and smoking across different parts of the healthcare cost distribution, which would not have been revealed under a traditional mean-based approach such as GLM. As Figure 1 illustrates, the GLM- based effects of obesity and smoking can be substantially overestimated in the lower tail of the cost distribution, while they might be substantially underestimated in the upper tail of the cost distribution. Note that the heterogeneity of conditional and unconditional effects is the net result of the interaction between "within-group" and "between-group" effects.[21,44,48] In the specific application in our study, it turns out that the within- and between-group effects appear to move in tandem in the upper quantiles of the cost distribution, while it is opposite in the lower quantiles. This is evident from the fact that the estimated effects were substantially greater in the upper quantiles than those in the lower quantiles of healthcare cost distribution.

Employers are increasingly concerned with the impact on employee productivity and healthcare cost from known health risk factors. Obesity and smoking have been identified as two leading modifiable health risks with significant impact on healthcare costs.[15] This paper evaluated distributional impacts of smoking and obesity on overall healthcare cost. Understanding the impact of incremental costs of the upper quantile groups, as opposed to the standard mean-based analysis, may help develop more effective targeted worksite wellness programs. Knowledge of heterogeneous cost impacts from obesity and smoking, as shown in this study, could be the necessary impetus to justify even more mandated interventions.

**Potential Limitations**

Obesity and smoking were modeled as two independent predictors of healthcare cost. Our approach, at first glance, may appear at odds with some of the previous works in this area.[25,26,27] However, note that there is still considerable ambivalence in the literature on the dependence between obesity and smoking. For example, while Baum[27] finds significant positive association between smoking and obesity, Flegal[26] finds that the decrease in smoking prevalence is often associated with a less than 1% increase in the prevalence of obesity. Sturm[10] modeled obesity and smoking as two independent risk factors, as was implemented in our study. Chen *et al.*[28] suggest that smoking does not have a long-term causal effect on body weight and Nonnemaker *et al.*[29] refute the claim that reduction in smoking (through cigarette tax) is associated with an increasing trend in obesity. Although Gruber and Frakes[25] found that reduced smoking leads to lower body weight, they could not confirm this association conclusively.

In order to rule out the possibility that the potential dependence between obesity and smoking unduly bias the results, we conducted several sensitivity analyses, described in detail in Section 3. We included interactions between obesity and smoking in the models but the corresponding coefficients were not significant in UQR and CQR for most of the quantiles. Thus, we are confident that any bias in the effect estimates due to modeling obesity and smoking as separate covariates is negligible.

Education and socioeconomic status have been found to be associated with obesity and smoking.[49,50,51,52]

Note, however, that the exact mechanism through which education impacts health is still inclusive.[49] Thus, our approach of controlling for education level as an independent predictor in UQR, CQR and GLM is aligned with the above finding. Our results suggest that higher education is negatively associated with healthcare costs. We controlled for race of the patient. However, our study sample was overly white (97%) and therefore race did not appear to have any independent effect on healthcare costs. Another measure of socioeconomic status, household income, was not available in the data. However, we believe that education level, which is adjusted for in the study results, is a good surrogate for income. Prior studies found income to be negatively associated with obesity.[53,54] We speculate that the omission of income in our study may under- or over-estimate the effects of obesity on healthcare cost depending on the average income of the study subjects.

The study used BMI as the measure of obesity despite the common criticism that BMI does not distinguish fat from fat-free mass such as muscle and bone.[55] As with other studies in the literature, we could not use more acurate measures of obesity because they were not available for the majority of study subjects. Another potential limitation of the study is the possibility of crossover of subjects in different smoking status or obesity categories between the baseline and the follow-up periods. However, our preliminary look at the available data shows that such crossover (e.g., from non-smoker to smoker, obesity to morbid obesity) during the course of the 5-year follow-up period is negligible. Therefore, we anticipate that our results are robust to the presence of such negligible crossover effects.

### CONCLUSION

This study applied a recently developed novel econometric technique, UQR, to assess the impacts of obesity and smoking on overall healthcare costs. The study results highlight the advantages of UQR over CQR, and the traditional approach of GLM used for healthcare cost modeling. The results suggest that the impacts of obesity and smoking were substantially higher in the upper tail of the cost distribution compared to those in the lower tail. The UQR results also demonstrate the heterogeneity of impacts of obesity and smoking across different parts of the cost distribution not captured by traditional mean-based approaches.

The findings of this paper have some important policy implications. While obesity and smoking have positive impacts on mean healthcare costs, the impact can vary substantially across the cost distribution. When planning wellness initiatives with the goal of reducing healthcare costs, employers will have to consider whether they will be able to target the right part of the spending distribution.

### ACKNOWLEDGEMENT

The authors thank Megan Branda and Amy E. Wagie of Division of Health Care Policy and Research, Mayo Clinic, for programming the analytic data file. The authors accept responsibility for the remaining errors.

### CONFLICT OF INTEREST DECLARATION

The authors declare that there are no competing interests.

## Figures and Tables

**Figure:** Supplementary Content

